# Metastatic Melanoma Mimicking Primary Breast Cancer—A Diagnostic Challenge: A Case Report

**DOI:** 10.1155/crip/8820195

**Published:** 2026-02-24

**Authors:** Angela de Salles Rezende, Teresa Cristina Ferreira Gutman, Marcus Vinicius Teixeira Calejon Stumpf, Karin Soares Gonçalves Cunha, Fabiana Resende Rodrigues, Vânia Gloria Silami Lopes

**Affiliations:** ^1^ National Cancer Institute (INCA), Rio de Janeiro, Rio de Janeiro, Brazil, inca.gov.br; ^2^ Department of Pathology, Gaffrée Guinle University Hospital (UNIRIO), Rio de Janeiro, Rio de Janeiro, Brazil; ^3^ Medical College, Fluminense Federal University, Niterói, Brazil, uff.br; ^4^ Department of Pathology, Fluminense Federal University, Niterói, Brazil, uff.br

**Keywords:** breast neoplasms, diagnosis differential, immunohistochemistry, melanoma, neoplasm metastasis

## Abstract

**Introduction:**

Metastatic melanoma to the breast is rare and may be misdiagnosed as primary breast carcinoma. Histological, immunohistochemical, and clinical correlations, such as a history of previous melanoma, are essential for diagnosis.

**Patient Presentation:**

A female patient presented with melanoma in the left gluteal region. Five years later, a palpable nodule was detected in the right breast on routine mammography. Microscopic and immunohistochemical examination confirmed the diagnosis of metastatic melanoma. The patient passed away 3 years later.

**Conclusion:**

The findings in this case indicate that metastatic melanoma can present in atypical ways and that specialists should pay attention to metastases in unusual locations in cases of melanoma.

**Summary**

• Established facts
o. Melanoma is associated with high morbidity and mortality.
• Novel insights
o. A case of cutaneous melanoma, which developed a rare breast metastasis clinically mimicking a primary breast tumor.

## 1. Introduction

Melanoma is a type of tumor that originates from melanocytes, and its incidence continues to increase, particularly in countries with fair‐skinned populations [1]. Globally, 324,635 new cases of skin melanoma and nearly 57,043 deaths are estimated to occur annually [2].

The most frequent extramammary tumors exhibiting breast metastases, in order of occurrence, are carcinoma (50%)—including those of ovarian, lung, gastrointestinal tract, kidney, thyroid, and head and neck—followed by melanoma (22%), sarcomas (20%), neuroendocrine tumors (4%), and lymphomas (4%) [3, 4].

Isolated axillary metastasis is observed in only 8% of patients, while simultaneous breast and axillary involvement occurs in 14% at the time of diagnosis [5]. Melanoma can have several presentations in the breast, such as primary skin melanoma, primary breast melanoma, melanoma metastasis, and in‐transit metastases (macroscopic lesions located more than 2.0 cm from the primary tumor or the lymphatic drainage or toward it) [6]. Melanomas metastasize in approximately 20% of cases. Breast metastases from malignant melanoma are rare, representing between 1.3% and 2.7% of all neoplastic manifestations in the breast [7]. It makes this case unique, and it provides a new perspective on the need to accurately differentiate breast melanomas from actual breast cancers.

Therefore, accuracy in the diagnosis of metastatic breast tumors is crucial because the staging, treatment, and prognosis of these tumors are essentially different from those of primary breast tumors. However, owing to the rarity and atypical histology of breast melanomas, obtaining an accurate pathological diagnosis is sometimes challenging. Certain clinical and histopathological features, such as a history of extramammary malignancy, unusual histology for primary breast cancer, atypically rapid growth, normal CA15‐3 levels, circumscribed tumors with multiple satellite foci, absence of intraductal components, numerous lymphovascular neoplastic emboli, lack of a breast‐related immunophenotype, and presence of an immunophenotype typical of an extramammary origin, are valuable for the diagnosis of metastasis to the breast [8, 9]. In addition to similar histology, the presence of brown cytoplasmic pigment, intranuclear inclusions, marked pleomorphism, and spindle cells, biomarkers can be evaluated when metastatic melanoma is suspected [10].

## 2. Case Presentation

A 56‐year‐old female patient presented with an extensive superficial ulcerated melanoma in the left gluteal region measuring 1.9 × 1.8 *c* *m*. Her past medical, family, and psychosocial histories were otherwise unremarkable, with no reported family history of cancer or known genetic syndromes. The histopathological exam demonstrated classic histomorphologic features that provide diagnostic clues for malignant melanoma, such as melanin pigment, nuclear pseudoinclusions, prominent nucleoli, eccentric nuclei, and discohesive cells. A biopsy performed 8 years ago revealed a melanoma with a Breslow thickness of 2 mm, presence of ulceration, 11 mitoses/mm^2^, and Clark Level III. The tumor was located 0.5 cm from the nearest lateral resection margin and 2.5 cm from the deep margin. The lesion exhibited an extensive “in situ” melanoma component, lymphovascular neoplastic emboli, perineural neoplastic infiltration, microsatellitosis, and solar elastosis. A focal and inactive lymphocytic inflammatory infiltrate (nonbrisk) was present. No areas of regression or ulceration were observed. Enlargement of the surgical margins, which were free of residual neoplasia, was performed 1 month after, and scar fibrosis, foreign body–type giant cell reactions, and steatonecrosis were observed.

Four years after the initial diagnosis, the patient presented with a nodule in the right axilla measuring 1.4 × 1.0 *c* *m*, which was surgically removed. The histopathologic exam demonstrated clusters of undifferentiated cells, typically with extreme cytologic atypia and pleomorphism, with very large, irregularly shaped, brightly eosinophilic nucleoli and intranuclear inclusions. Microscopic differential diagnoses were considered carcinoma and lymphoma. Nuclear features, such as nuclear inclusion and the presence of cytoplasmic pigment, indicated the diagnosis of melanoma. The immunohistochemical examination (HMB‐45 and MART1/Melan‐A) confirmed the diagnosis of metastatic melanoma.

**Figure 1:**
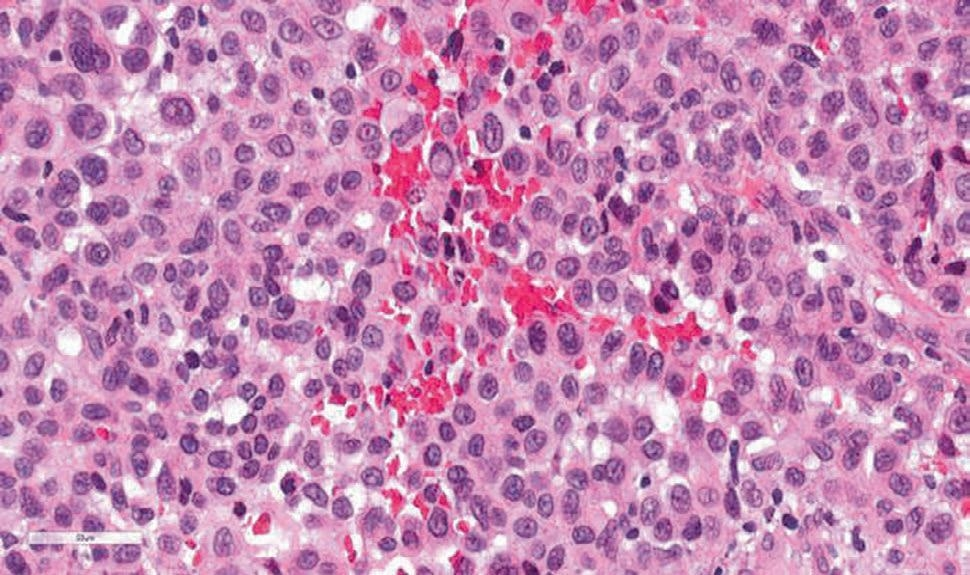
Hematoxylin and eosin–stained section of metastatic melanoma in the breast parenchyma. Tumor cells with epithelioid morphology, nuclear pleomorphism, hyperchromasia, prominent nucleoli, and mitoses are shown (H&E, 40×).

**Figure 2:**
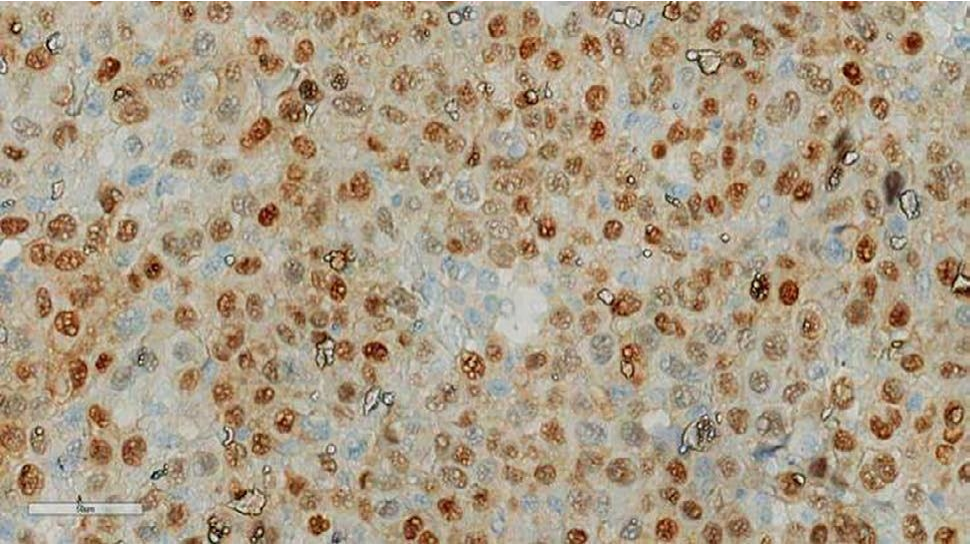
MART1/Melan‐A staining demonstrated a top‐heavy cytoplasmic and nuclear staining profile within malignant cells IHC, 40×.

Five years after the initial melanoma diagnosis, a nonpalpable, solid‐cystic, dense, regular, and circumscribed nodule, measuring 1.7 × 1.5 *c* *m*, located 6.7 cm from the nipple in the anterior third of the upper quadrant of the right breast, was detected on routine mammography. Breast ultrasound was performed, and the lesion was classified as BI‐RADS Category 4. No additional lesions were detected in the right or contralateral breast. No axillary lymph nodes were visualized. PET‐CT scan demonstrated hypermetabolic activity in the breast compatible with diagnosed melanoma. A lumpectomy with clear surgical margins was performed. The axillary sentinel lymph node was negative for metastasis. Histopathological examination showed that the cells were similar to those found in the nodule in the armpit about a year ago. Tumor cell islands exhibit epithelioid morphology, wide eosinophilic cytoplasm, intracytoplasmic (brown) pigment accumulation, nuclear pleomorphism, prominent eosinophilic nucleoli and intranuclear inclusion, and necrosis. Immunohistochemistry (IHC) evaluation was positive for antibodies against S100, HMB‐45, and MART1/Melan‐A; negativity for antibodies against AE1/AE3 and LCA confirmed the presence of metastatic melanoma (Figures 1 and 2).

Three years after this last diagnosis, the patient developed a metastatic lesion in the frontal region of the brain. The mass measured 3.8 cm on its largest axis, with a mass effect and midline shift, along with signs of recent bleeding. The lesion with well‐demarcated margins and dark color was resected. Immunohistochemical melanocytic markers, including S100, Melan‐A (MART1), and HMB‐45, were positive, supporting the diagnosis of metastatic melanoma. Following the diagnosis of metastatic disease, the patient received appropriate adjuvant and metastatic immunotherapy according to the standard‐of‐care protocols available at the time.

The patient experienced a seizure in the immediate postoperative period which was treated. She developed progressive and life‐threatening organ failure within a week.

## 3. Discussion

This case report provides a detailed account of a rare diagnostic challenge; however, its findings must be interpreted considering its specific methodological framework. The principal strengths of this report include the detailed longitudinal follow‐up over an 8‐year clinical course and the comprehensive histopathological and immunohistochemical analysis that was critical for accurate diagnosis. The main limitations are inherent to the study design: As a single, retrospective case report, the findings are not generalizable and serve primarily to illustrate a diagnostic pitfall and support existing literature.

The presentation of a breast nodule in perimenopausal women is often initially considered a primary malignancy because of the rarity of metastasis to the breast from extramammary sites [11]. In patients with known malignancies, the possibility of breast metastases should be considered, even when clinical findings appear benign, as treatment and prognosis differ significantly from those of primary breast cancer. Malignant melanoma can mimic many types of poorly differentiated tumors, which makes its diagnosis challenging, especially when it presents as an isolated breast tumor [12]. In contrast to primary breast cancer, breast metastases typically do not demonstrate spiculated margins, skin involvement, or nipple retraction [13].

Immunohistochemical examination plays an important role in distinguishing secondary from primary breast cancer, especially in patients with a tumor history [14]. The absence of immunostaining for pancytokeratin may suggest that the breast tumor under analysis is extramammary in origin. The S100 is a sensitive marker for melanoma, but it can mark some types of breast carcinoma. For this reason, a sensitive and specific IHC panel must be used [15].

Metastatic melanoma presents as multiple lesions rather than a solitary mass, as observed in the brain in our case [16].

Primary melanomas located at the Achilles region, upper arms, and face have been associated with better prognosis. In contrast, melanomas arising on the middle and lower back, as well as in supramammary and mammary regions, are independent prognostic factors for poor outcomes [17]. In our case, the primary melanoma was located on the left gluteal region, while the axillary mass was on the right side, raising uncertainty as to whether the axillary lesion represented a primary or a metastasis, and, consequently, where the true primary site was. The site of the first metastasis appears to be the most important prognostic factor for survival after recurrence (Ramos [18]). At the same time, the presence of melanoma metastasis indicated systemic dissemination and a more aggressive disease course. In agreement with the literature, the first diagnostic metastasis in our case was in the axilla. But, similar to our case, the average interval between the primary diagnosis and breast involvement was 5 years, and the median survival after the diagnosis of breast metastasis is 1 year [19].

Histopathological characteristics that indicate a potentially worse prognosis include older age, location (acral, head, and neck), male sex, increased tumor thickness (Breslow), increased anatomical level (Clark), ulceration, increased number of mitoses, vertical growth phase, regression, absence of a host inflammatory response, increased tumor vascularization, angiotropism, vascular invasion, neurotropism, marked atypia, and satellite metastases [20]. Among these factors, three histopathological characteristics of primary melanoma are currently the most important prognostic and staging factors: Breslow tumor thickness, mitotic rate, and the presence or absence of ulceration [21].

Metastatic malignant melanoma has an extremely poor prognosis [22]. Once the first distant metastasis appears, the disease becomes more aggressive [23]. For prevention of recurrence, due to the uncertain prognosis, complete surgical excision and a yearly clinical follow‐up are recommended. This approach was followed in the case of brain metastasis. In the central nervous system, much more frequently in the intracranial space, multiple metastatic spread is most common from malignancy originating from another organ (e.g., lung, breast, and malignant melanoma) [24], although a solitary metastatic spread can occur, as in our case.

The estimated 5‐year survival rate for patients with metastatic melanoma is 5.2% [25]. Similar to our case, the median survival time for patients with multiple melanoma metastases is less than 1 year [26].

Metastatic tumors in the breast represent a diagnostic challenge for both clinicians and pathologists. Histological clues may facilitate diagnosis and may include the presence of histological pigment, plasmacytoid features, the presence of previous neoplasia in another location, and the absence of carcinoma in situ; the metastatic disease to the breast parenchyma is considered, but none of these is specific. Careful correlation with the clinical history and an immunohistochemical panel can assist in making the final diagnosis.

As the patient was deceased at the time of manuscript preparation, her personal perspective on the treatments received could not be obtained.

## 4. Conclusion

This case highlights the critical role of a multidisciplinary approach in unraveling the complexities of metastatic melanoma, where clinical and histopathological aspects converge for an accurate diagnosis.

## Funding

No funding was received for this manuscript.

## Ethics Statement

This work was approved by the Research Ethics Committee of Fluminense Federal University (CAAE 43900620.8.0000.5243). This research standard is defined in Resolution National Health Commission (CNS) No. 466 of 2012 and in Operational Standard No. 001 of 2013 of the CNS.

## Consent

As the patient is deceased, consent for publication was obtained from his next of kin.

## Conflicts of Interest

The authors declare no conflicts of interest.

## Data Availability

The data that support the findings of this study are not publicly available and were used under license for the purpose of the research. However, it can be accessed upon reasonable request and with permission of the first author.
